# High-Sensitivity Flexible Pressure Sensor-Based 3D CNTs Sponge for Human–Computer Interaction

**DOI:** 10.3390/polym13203465

**Published:** 2021-10-09

**Authors:** Jianli Cui, Xueli Nan, Guirong Shao, Huixia Sun

**Affiliations:** 1 Department of Physics and Electronics Engineering, Yuncheng University, Yuncheng 044000, China; shaoguirong@sina.com (G.S.); 13389266290@163.com (H.S.); 2 Department of Measurement & Control Technology and Instrument, Shanxi University, Taiyuan 030006, China

**Keywords:** CNTs sponge, CVD, pressure sensor, high sensitivity, human–computer interaction

## Abstract

Researchers are showing an increasing interest in high-performance flexible pressure sensors owing to their potential uses in wearable electronics, bionic skin, and human–machine interactions, etc. However, the vast majority of these flexible pressure sensors require extensive nano-architectural design, which both complicates their manufacturing and is time-consuming. Thus, a low-cost technology which can be applied on a large scale is highly desirable for the manufacture of flexible pressure-sensitive materials that have a high sensitivity over a wide range of pressures. This work is based on the use of a three-dimensional elastic porous carbon nanotubes (CNTs) sponge as the conductive layer to fabricate a novel flexible piezoresistive sensor. The synthesis of a CNTs sponge was achieved by chemical vapor deposition, the basic underlying principle governing the sensing behavior of the CNTs sponge-based pressure sensor and was illustrated by employing in situ scanning electron microscopy. The CNTs sponge-based sensor has a quick response time of ~105 ms, a high sensitivity extending across a broad pressure range (less than 10 kPa for 809 kPa^−1^) and possesses an outstanding permanence over 4000 cycles. Furthermore, a 16-pixel wireless sensor system was designed and a series of applications have been demonstrated. Its potential applications in the visualizing pressure distribution and an example of human–machine communication were also demonstrated.

## 1. Introduction

Flexible pressure sensors have attracted great interest with the expeditious advancement in the realm of wearable electronics. This is owed to their prospective uses in biomedical monitoring [1,2,3,4], electronic skin [5,6,7,8], motion detection, and human–machine interactions [9,10,11], etc. The realization of flexible sensing technology has subverted people’s cognition about the form and function of traditional sensor devices. There are, in general, four categories of pressure sensors according to the differences in their functioning mechanisms. These categories include piezoelectric [12], capacitive [13], triboelectric, and piezoresistive [14,15] types. Among these, piezoresistive sensors have been characterized by their capacity to convert the pressure exerted upon them into a change in resistance or current and have been explored quite extensively. Of the number of advantages offered by piezoresistive sensors, a few include the simplicity of the fabrication process, high sensitivity, quick response time, and low energy consumption. Microstructure design, for instance, hollow-sphere microstructure, porous structure, interlocked microstructures, fractured microstructure, and micro pyramid array, are usually recognized as useful techniques for the fabrication of pressure sensors. However, the majority of these microstructure designs are difficult to manufacture, and the technology involved is exorbitant and complex [16,17]. Hence, until now, large-scale, low-cost fabrication of sensitive pressure sensors has been quite challenging [18,19,20].

Next-generation electronic devices bear stretchable conductive materials (SCMs) [21,22] as a major key component, representing one of the most widely investigated areas in materials science and engineering. SCMs have significant benefits associated with them in comparison with traditional conductors, particularly with regard to their high conductivity, large-strain durability (>1%), and outstanding capability to retain conductivity during repeated deformation. Some sponge-like structures with highly porous and conductive surfaces are available for piezoresistive sensors. It is, however, unfortunate that these pristine porous structures have some associated shortcomings. They tend to collapse when subjected to large compressive stress owing to their poor mechanical properties. Meanwhile, most of those reported sponge-like materials enable piezoresistance by a physical coating of conductive materials, but the conductive materials can easily fall off during compression. Porous CNTs sponges [23,24,25], which are obtained via direct synthesis by chemical vapor deposition (CVD) [26], are classic SCMs for flexible pressure-sensitive conductive elastic fillers because of their good elasticity and exceptional electrical conductivity.

This work describes a flexible piezoresistive sensor constructed from a CVD-grown CNTs sponge with interdigital electrodes as well as large linearity and ultra-high sensitivity. Scanning electron microscopy (SEM) studies conducted in situ demonstrate that under the influence of external force, the CNTs sponge microfibers twist and contort and come into contact with one another. Consequently, at the contact points among the CNTs microfibers, fresh conductive pathways are formed, providing the fundamental sensing philosophy defining the piezoresistive sensor. The sensor manifests reliability under repeated stress and reflects both small and large compression strains accurately. In addition, we investigated its prospective applications in the measurement of human–computer interaction and pressure distribution.

## 2. Experimental Section

### 2.1. Materials

99.5% Ferrocene (C_10_H_10_Fe) was procured from Alfa Aesar. PDMS elastomer (SYLGARD 184) was obtained from Dow Corning Co., Ltd. (Shanghai, China). 99.9% pure 1,2-dichlorobenzene (C_6_H_4_Cl_2_) was bought from Sinopharm Chemical Reagent Co. Ltd. (Shanghai, China). The interdigital electrode (5 mm × 5 mm, line width 100 μm, line space 100 μm) was acquired from Shangyou Electronic Technology Co., Ltd. (Shanghai, China). Analytical-grade reagents were used in all experiments. The argon (Ar) employed for CNTs sponge growth was ultra-high purity (99.999%).

### 2.2. Synthesis of 3D CNT Sponge

A thermal CVD method was used to synthesize the CNTs sponge, using a horizontal resistance furnace. The carbon source and catalyst precursor employed were 1, 2-dichlorobenzene and ferrocene, respectively. A measure of 50 mL of 1,2-dichlorobenzene was used to dissolve 3 g of ferrocene completely using ultrasound, thus obtaining a 0.06 g mL^−1^ carbon source solution. The reaction zone temperature was set at 870 °C and the preheating of the feed was set to 250 °C. The carbon source was introduced via a branch piping located in the preheating region into the quartz tube once a stable temperature was attained in the reaction zone. For this purpose, a precision feed pump was employed. The experiment was carried out at a feed rate of 0.125 mL min^−1^. A mixture of H_2_ and Ar and was used as the carrier gas with their flow velocities maintained at 300 mL min^−1^ and 2000 mL min^−1^, respectively. The carrier gas mixed with the carbon source was pushed into the reaction zone to enable the carbon nanotubes to undergo catalytic growth. Intertwined carbon nanotubes fluctuated with the airflow and aggregated into the 3D macroscopic CNTs sponge after deposition on the quartz boat, which was located behind the reaction region.

### 2.3. Characterization of CNT Sponge

SEM (Hitachi S-4800) was used to characterize the CNTs microstructure and macroscopic sponge morphology. A Raman spectrometer (Renishaw inVia plus) was used to record the Raman spectra at an excitation wavelength of 532 nm (Thermo Electron, DXR Microscope).

### 2.4. Force Response Measurement

To test the CNTs sponge-based sensor in terms of its sensing performance under the influence of outside forces, a computer servo-controlled vertical pressure-testing machine (HD-B609B-S) was employed for uniaxial compression and release. A SourceMeter (Keithley 2450) was used to measure the current of the sensor under various pressures. When testing, the input voltage was maintained at a value of 1 V.

### 2.5. Wireless Detection of Motion

The sensor based on the CNTs sponge was allowed to come into contact with the joint or muscle after being wired to the pre-manufactured printed circuit board. A digital signal was obtained after the conversion of the response of the sensor, regulated via an Arduino Nano, and transmitted via the HC-05 Bluetooth module to a smartphone. Real-time monitoring of human motion was achieved via an app developed by Kivy.

## 3. Results and Discussion

**Figure 1:**
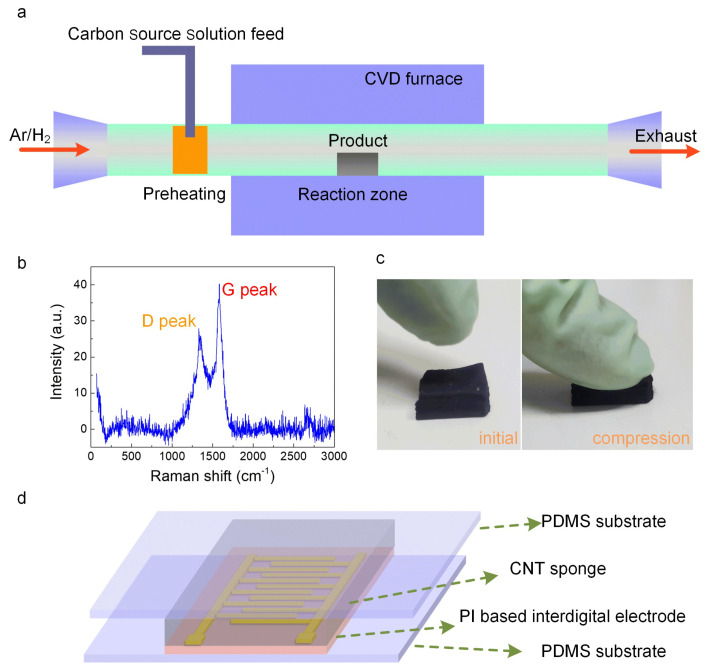
(**a**) Diagrammatic representation of the device employed for synthesizing the CNTs sponge using the CVD technique. (**b**) Raman spectra of the CNTs sponge. (**c**) Photographic macroscopic images of the CNTs sponge before and after compression. (**d**) The schematic representation of the CNTs sponge-based flexible pressure sensor.

Figure 1a schematically illustrates the synthesis process which takes place in a horizontal resistance furnace using the thermal CVD method. Catalytic pyrolysis of the carbon source, 1,2-dichlorobenzene, using ferrocene as the catalyst resulted in the successful synthesis of CNTs. Raman spectroscopy (Renishaw inVia plus) can be employed to identify the degree of crystallization of the CNTs sponge, as shown in Figure 1b. The Raman spectrum depicts two distinct characteristic peaks: one is the defect peak (D peak), while the other is the graphite peak (G peak). The D peak, representing lattice defects, primarily comprised of five-membered rings, seven-membered rings, and boundaries resulting from carbon atoms, can be seen at ~1340 cm^−1^. The G peak arising from the sp^2^ hybridization of carbon atoms is located at ~1579 cm^−1^. The D peak was notably lower in intensity in comparison to the G peak, thereby implying that the crystallization of CNTs was adequately accomplished. The outstanding compressibility and restorability of the conductive CNTs sponge are very well demonstrated by the initial and compression processes (Figure 1c) and were due to its highly porous nature. Thus, it is evident that the CNTs sponge has an immense capacity to function as variable conductors in human motion detection and artificial intelligence-related applications, possessing an elaborate domain of compression pressure/strain sensing abilities. Figure 1d depicts the schematic diagram of the CNTs sponge-based pressure sensors. The encapsulation layer for the bottom and top substrates was chosen to be polydimethylsiloxane (PDMS). Additionally, the biocompatibility, massive elasticity, as well as exceptional optical characteristics of PDMS have made it the most effective material for fabricating flexible electronic skin. CNTs sponge with a 5 × 5 × 2 mm block size was chosen as the intermediary conductive elastic filler. The reception of varying electrical signals was made possible by the use of the commercial rectangular interdigital electrode based on polyimide (PI), which is characterized by its ideal durability, tensile strength, and associated mechanical properties. Thus, the interdigital electrode was connected at both ends using silver paste with two copper wires to create the external contact.

**Figure 2:**
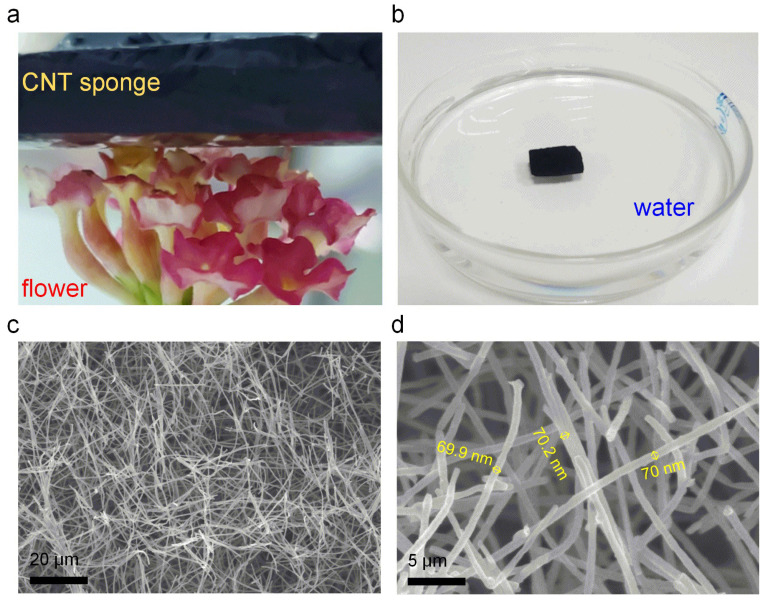
(**a**) Photograph of 3D CNTs sponge placed on top of a flower. (**b**) Photograph of 3D CNTs sponge on the water. (**c**,**d**) SEM photographs of the CNTs sponge comprising CNTs. The CNTs are ~70 nm in diameter.

The CNT sponge is characterized by an ultra-low density, reflected by the fact that a 5 cm^3^ mass of this CNT sponge can be held upon the florets of a flower head with no visible distortion (Figure 2a). Moreover, the CNT sponge can float completely above water, which is additional proof of its ultra-low density (Figure 2b). To acquire a thorough understanding of the microstructure of CNT sponge, a scanning electron microscope (SEM) was used to observe the agglomeration of CNTs in the sponge. Figure 2c,d, respectively, depict the microscopic structural features of CNTs sponge at a low and high power of magnification. As evident in Figure 2c, carbon nanotubes are interwoven into a network structure with very high voltage. Simultaneously, it can be seen that the transverse and longitudinal aspects of carbon nanotube sponges are not different, and rather have isotropic characteristics. The uniformity of the diameter of CNTs is demonstrated via the high magnification SEM images, as shown in Figure 2d. The diameter of CNTs is ~70 nm.

**Figure 3:**
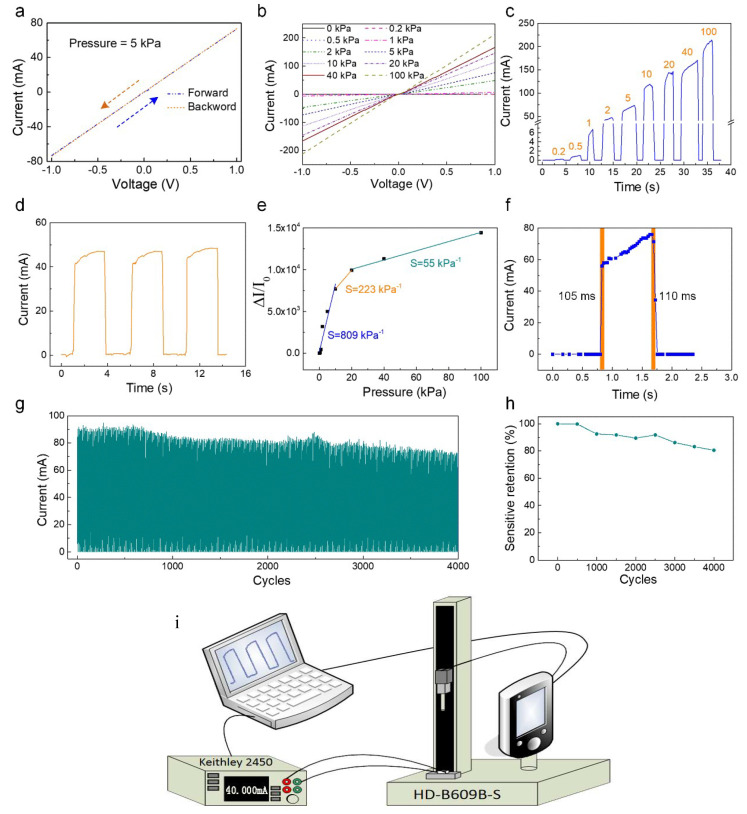
(**a**) I-V measurements at an external pressure of 5 kPa for forward and backward sweepings of voltages from −1 to 1 V. (**b**) The straight-line relationship between the I–V curves for the voltage from −1 to 1 V. (**c**) The I–T curves of the pressure sensor with the various external forces. (**d**) The stability of the pressure sensor. (**e**) The sensitivity of the sensor, demonstrating a high sensitivity of 809 kPa^−1^ under 10 kPa, 223 kPa^−1^ in the region of transitional pressure (10–20 kPa), and 55 kPa^−1^ in the region of high-pressure (20–100 kPa). (**f**) The response time in relation to the loading and unloading pressure of the sensor. (**g**) The durability of this sensor with press-release 4000 cycles. (**h**) The sensitivity remained well above 80% of the original value, following 4000 loading–unloading cycles. (**i**) Schematic illustration of the test system.

The piezoresistive influence of the sensor was investigated by examining the electrical response of the instrument at various values of pressure using a complete system for testing electrical signals. As presented in Figure 3i, the test system consisted of an electrical signal processing device of SourceMeter (Keithley 2450), a computer servo-controlled vertical pressure-testing machine (HD-B609B-S). The above piezoresistive sensor was placed at one end of the vertical pressure-testing machine with its electrodes connected to the electrical signal processing device. The signal processing device converts the mechanical signal into a corresponding electrical signal while exerting an external force on the sensor. Voltages from −1 to 1 V were used to measure the forward and backward sweeping I–V curves of the designed sensor at an outside pressure (Figure 3a). The forward sweeping I–V curves coincide with the backward curves. This implies an excellent Ohmic contact of the CNTs sponge with the interdigital electrodes. In the voltage range from −1 to 0.1 V, the linearity of the relationship of the I–V curves (Figure 3b) demonstrated that an ohmic contact was created between the interdigital electrode and the CNTs sponge. With the increment in the applied load, there was a corresponding increase in the slope of the I-V curve, thereby demonstrating that the sensor of the multilayer CNTs sponge accordingly manifested a decrease in its resistance. There was a monotonic increment in current as the pressure increased, shown by the I–T curves (Figure 3c). This suggests that the designed pressure sensor can distinctly identify differing degrees of external force. Moreover, Figure 3d depicts the high stability of the pressure sensor. The sensitivity and responsiveness of a piezoresistive device is a notable variable for determining the performance of the device and is in general defined by the expression *S* = (*ΔI*/*I*_0_)/*ΔP*, where the difference in the current before and after subjecting to pressure is denoted by *ΔI*, and the initial value of current before the application of pressure is represented by *I*_0_. The magnitude of change in pressure from *I*_0_ to *I* is represented by *ΔP*. Figure 3e represents the estimation of *ΔI*/*I*_0_ relative to the pressure value. The outcome demonstrates that there are three regions in which the current curve varies linearly with the variation in pressure. The value of the sensitivity of the instrument was as high as 809 kPa^−1^ in the low-pressure region (0–10 kPa), while in the transitional pressure region (10–20 kPa) and high-pressure region (20–100 kPa), the sensitivity of the device was 223 kPa^−1^ and 55 kPa^−1^, respectively. Additionally, we estimated the response and recovery time of the instrument under the influence of pressure with results of 105 ms and 110 ms, which enabled testing in real time (Figure 3f). Further evaluation of the operational life and mechanical durability of the sensor was carried out by testing 4000 cycles of loading and unloading pressures, as depicted in Figure 3g. The sensitivity remained well above 80% of the primary value after 4000 loading–unloading cycles (Figure 3h).

**Figure 4:**
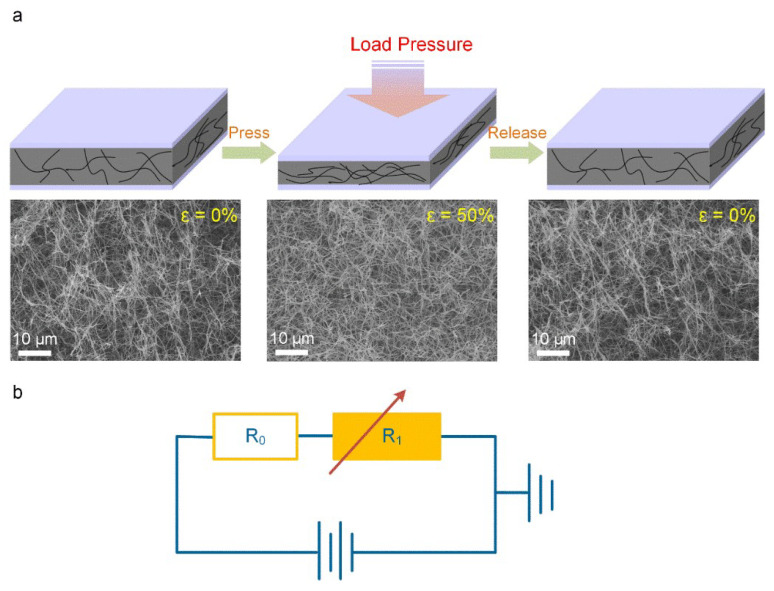
(**a**) Pressure sensing models of the synthesized CNTs sponge-based sensor under the press-release condition depicts the variation in the contact area of the CNTs network taking place with the compression deformation. The in situ SEM photographs of the CNTs sponge press-release dynamic process: The CNTs sponge in the original state (left), pressed state of ~50% (center), after releasing completely (right). (**b**) An equivalent circuit representation of the piezoresistive sensor based on CNTs sponge, where *R*_1_ is ascertained by the internal voids distance of the CNTs sponge whereas *R*_0_ has a constant value.

The underlying principle governing the sensing function of the CNTs sponge-based sensor is depicted in Figure 4a. The linear stretch between the internal voids of the CNTs sponge reduced following the application of external pressure. Since there is close contact between the CNTs, there is a consequent increase in the contact. region of the CNTs matrix, as well as in the resistance of the CNTs sponge *R*_1_ (relevant to the CNTs sponge’s integral resistance). In contrast, the deformed CNTs are re-established and the contact area of the CNTs network is reduced upon the release of the loading pressure of the sensor. In situ SEM demonstrates the real-time change in the microstructural framework of a CNTs sponge under the influence of various compression conditions. In comparison with the original CNTs sponge, there was a significant reduction in the size of the macropore of the compressed CNTs sponge. Remarkably, upon the release of the applied pressure, the CNTs sponge almost completely restored its original shape. This is further evidence of the excellent reusability of the pressure sensor based on CNTs sponge. The equivalent equation *R*_Total_ = *R*_0_ + *R*_1_, wherein the total resistance is represented by *R*_Total_ and the contact resistance between the interdigital electrode and the CNTs sponge is represented by *R*_0_ (Figure 4b). *R*_0_ primarily remains unaltered because it is expected to behave independently of the pressure applied. Hence, only the change of *R*_1_ is responsible for the value of *R*_Total_, which is the piezoresistive characteristics of the CNTs sponge.

**Figure 5:**
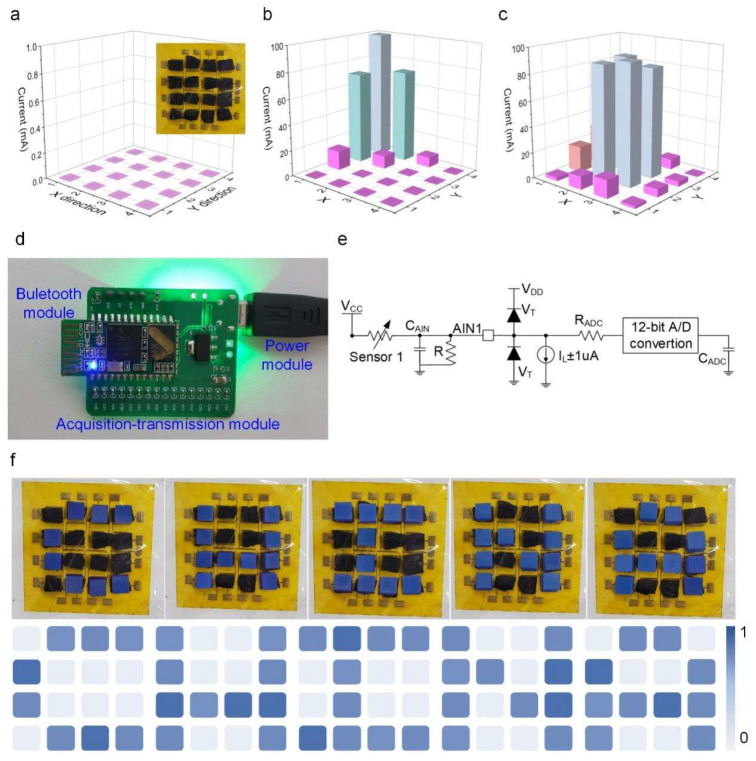
(**a**) The initial response of the 16-pixel pressure sensor array. (**b**,**c**) The current response of the pressure sensor array when pressed upon different positions on the device. (**d**) The physical diagram of the printed circuit board. (**e**) Block diagram of the readout circuit. (**f**) Different pressure graphics: physical map and terrain recognition normalized demonstration results.

To demonstrate the prospective uses of the designed sensor, we fabricated a 16-pixel (4 × 4 elements) pressure sensor array to detect the pressure distribution, as shown in the inset of Figure 5a. When no pressure is applied to the sensor array, the calibration color of each sensor is purple. With the application of pressure upon different positions on the sensor array, there is a change in the color of the corresponding sensor owing to the different amounts of force loaded on each sensor, as shown in Figure 5b,c. The outcome demonstrates the excellent sensitivity of the sensor array to various outside forces. These results prove that sensors based on CNTs sponge can be successfully applied to tactile detection and mechanical image recognition.

To probe into the prospective uses of the CNTs sponge-based sensor for utilization in the human–machine interface, we proceeded to design a wireless detection system. The system chiefly comprised; the acquisition–transmission module, the sensing module, and the display module. Figure 5d depicts the photograph of the printed circuit board. A fixed resistance (*R*), useful for avoiding short circuit and a filter capacitance (*C_AIN_*) for isolating the noise associated with AC, comprised the voltage dividing circuit unit. A Ti_2_C-PDMS sponge-based sensor was coupled with it, which was assumed to behave as a variable resistor. The A/D signal acquisition component is an integral feature of the processor (MCU). A pair of clamping diodes (*V_T_*), a sampling switch resistor (*R_ADC_*), a filtering capacitor (*C_ADC_*), and a 12-bit A/D converter together constituted an A/D acquisition circuit. The digital responses corresponding to the force stimulation were transferred using a wireless technique in real time via the universal synchronous/asynchronous receiver/transmitter (USART) belonging to the HC-05 Bluetooth unit. Figure 5e shows the elaborate block diagram of the readout circuit.

Using the above-mentioned wireless human–computer interaction system as a model, we made use of the sensor array to demonstrate the process of topographical map recognition, as illustrated in Figure 5f. The letter was shaped using small iron cubes (5 × 5 × 5 mm^3^). The normalized image results show that the sensor array constituted from the CNTs sponge array can efficiently identify different shapes, such as C, H, I, N, A. Moreover, this display proved that the piezoresistive sensor comprised of CNTs sponge has a potential application value in several fields including human–computer interaction and wearable devices, etc.

## 4. Conclusions

The chemical vapor deposition method was employed for the synthesis of a 3D elastic porous CNTs sponge, which was then used in the piezoresistive sensor. It was illustrated intuitively by an in situ SEM-based investigation that under the influence of external force the microfibers of the CNTs sponge undergo distortion upon contact with one another. Consequently, new conductive pathways come into being at the point of contact between the microfibers of CNTs, which essentially shapes the fundamental theory behind the sensing activity for our piezoresistive sensor. The sensor based on CNTs sponge possesses a high sensitivity over a broad range of pressures (less than 10 kPa for 809 kPa^−1^), a quick response equivalent to a duration of ~105 ms, and outstanding longevity over a count of 4000 cycles. Keeping in view the mentioned sensing indicators of the sensor based on CNTs sponge, a wireless sensor system comprising 16 pixels has been successfully designed and manifested several useful applications.
